# Coordination Cost and Super-Efficiency in Teamwork: The Role of Communication, Psychological States, Cardiovascular Responses, and Brain Rhythms

**DOI:** 10.1007/s10484-020-09479-8

**Published:** 2020-06-19

**Authors:** Ben Hoyle, Jamie Taylor, Luca Zugic, Edson Filho

**Affiliations:** 1 School of Psychology, University of Central Lancashire, Darwin Building 114, Preston, PR1 2HE UK; 2 Social Interaction and Performance Science (SINAPSE) Lab, University of Central Lancashire, Preston, UK

**Keywords:** Group dynamics, Team coordination, Heart rate variability, EEG

## Abstract

To advance knowledge on the psychophysiological markers of “coordination cost” in team settings, we explored differences in meta-communication patterns (i.e., silence, speaking, listening, and overlap), perceived psychological states (i.e., core affect, attention, efficacy beliefs), heart rate variability (i.e., RMSSD), and brain rhythms (i.e., alpha, beta and theta absolute power) across three studies involving 48 male dyads (Mage = 21.30; SD = 2.03). Skilled participants cooperatively played three consecutive FIFA-17 (Xbox) games in a dyad against the computer, or competed against the computer in a solo condition and a dyad condition. We observed that playing in a team, in contrast to playing alone, was associated with higher alpha peak and global efficiency in the brain and, at the same time, led to an increase in focused attention as evidenced by participants’ higher theta activity in the frontal lobe. Moreover, we observed that overtime participants’ brain dynamics moved towards a state of “neural-efficiency”, characterized by increased theta and beta activity in the frontal lobe, and high alpha activity across the whole brain. Our findings advance the literature by demonstrating that (1) the notion of coordination cost can be captured at the neural level in the initial stages of team development; (2) by decreasing the costs of switching between tasks, teamwork increases both individuals’ attentional focus and global neural efficiency; and (3) communication dynamics become more proficient and individuals’ brain patterns change towards neural efficiency over time, likely due to team learning and decreases in intra-team conflict.

Different theoretical frameworks have been used to study teamwork across domains. From an evolutionary perspective, teamwork allows for *super-efficiency* in the natural world (Anderson and Franks 2001). That is, the outputs of teamwork are often greater than the sum of individuals’ outputs, akin to the gestalt notion that “the whole is greater than the sum of its parts”. Super-efficiency occurs because teamwork allows for adaptive specialization or division of labour (Duarte et al. 2012). Indeed, research has shown that social insects (e.g., ants and bees) rely on division of labour to generate greater outputs (Anderson and Franks 2001), geese migrate in flocks to conserve energy by catching each other’s updrafts (Weimerskirch et al. 2001), and wolf and lion packs engage in adaptive specialization (e.g., stalking and ambush) to take down large prey (Gable et al. 2018; Stander 1992). For humans, mega-projects, such as the international space station, would not be feasible without teamwork.

On the flip side, there is a cost to teamwork, often referred to as “coordination cost” (Becker and Murphy 1992). To be coordinated in space and time, teammates must invest time and energy to learn their distinct roles within the team (see Eccles, 2010), while also developing social bonds and trust with one another (Cooke et al. 2000; Filho 2019). In fact, congruent with the notion of *reciprocal determinism* put forth by Bandura (1997), team coordination influences and is influenced by team performance and other team processes, such as cohesion and collective efficacy (Bonebright 2010; Filho et al. 2015a, b; Gabelica et al. 2016; Mathieu et al. 2000). More centrally, coordination is thought to depend upon teammates’ shared and complementary knowledge types (i.e., knowledge of what, why, how, when and where; see Filho and Tenenbaum 2020). To this extent, multi-person physiological studies with interactive jugglers (e.g., Filho et al. 2015a, b; Filho et al. 2016; Filho et al. 2017; Stone et al. 2019) and duet-guitar players (see Sänger et al. 2012, 2013) have revealed that team coordination is possible because teammates activate shared and complementary brain areas to sustain joint attention and action. For effective coordination to occur in the natural world, teammates must (a) share knowledge about each other, the task, the “team as a whole” and the context; and (b) possess complementary knowledge that allows for the resolution of complex problems that hinge on “distributed cognition” (Cooke et al. 2000; Filho 2019; Filho and Tenenbaum 2020).

The higher the quantity and quality of teammates’ shared and complementary knowledge, the higher the chance the team as a whole will show optimal coordination (Gabelica et al. 2016; Mathieu et al. 2000). The development of team knowledge decreases coordination cost because teammates learn to communicate more effectively and save energy through the division of labour (Eccles 2010; Filho and Tenenbaum 2020). Indeed, research across domains, including studies with special police units (Boulton and Cole 2016), emergency medical teams (Westli et al. 2010), hand-to-hand circus acrobats (Filho and Rettig 2018), and interactive team sports (LeCouteur and Feo 2011), has revealed that over time teammates’ move from overt to covert communication and learn to synchronise their actions.

Noteworthy, the bulk of research on team coordination thus far has been primarily field-based, as applied psychologists are mainly interested in capturing team dynamics in situ (Filho and Tenenbaum 2020; Mohammed et al. 2010, 2017). To advance understanding of the psycho-bio-social mechanisms underpinning coordination cost, we conducted three experimental studies to explore changes in communication patterns and psycho-bio-social states within teams over time (Study 1 and 3), and between individual work and teamwork (Study 2). Theoretically, our work was grounded on the aforementioned notion that teamwork allows for super-efficiency. Methodologically, we used a video game setting, which has been deemed a reliable and ecologically valid experimental platform, to study socio-cognition in general (Gray 2017), and teamwork in particular (Galantucci 2005). Furthermore, we adopted a multi-modal approach given that team processes possess several psycho-bio-social markers or reflective indicators (Cacioppo et al. 2007; Hannah et al. 2013), and akin to the importance of data triangulation to prevent common methodological biases in applied research (Podsakoff et al. 2003; Thorson et al. 2018).

## Study 1

Over three matches of a dyadic video game, we explored changes in performance, communication patterns, core affect (arousal and pleasantness levels), efficacy beliefs, attentional states, and cardiovascular responses. As team coordination and other team processes develop over time and as a sense of team evolves, teammates communicate better and show more positive affect, efficacy beliefs, and functional joint attentional patterns; and less physiological stress (see Boulton and Cole 2016; Filho et al. 2015a, b, 2016, 2017; Filho 2019; LeCouteur and Feo 2011; Mohammed et al. 2010; Stone et al. 2019). Accordingly, over the three matches, we expected to observe improvements  in performance, communication patterns, positive core affect, and efficacy beliefs, and a decrease in attentional levels and cardiovascular responses.

## Methods

### Participants

Forty-eight male participants were assembled into 24 dyads. This sample size was based on research suggesting that data for at least 15 teams should be collected to allow for reliable parameter estimation in group dynamics research (Kerkhoff and Nussbeck 2019). The participants were twenty years old on average (M = 20.41, SD = 1.89) and had at least 30 h of experience playing FIFA 17, which is generally considered enough practice to secure learning in a motor task (see Ericsson 1998).

### Measures

### Performance Data

Performance measures included *Total Points* (win = 3 points; draw = 1 point; loss = 0 points), *Ball Possession*, *Goal Differential*, and *Number of Fouls*, and were generated for every match by the video game software. All of these variables have been used as indicators of team performance (Lago-Ballesteros and Lago-Peñas 2010).

### Subjective Data

Single-item measures of core affect (arousal and pleasantness), attentional states and efficacy beliefs (self-efficacy and others’ efficacy) were used to gather the participants’ subjective psychological states throughout the experimental task. Single-item measures have been used in applied psychology, as they can be quickly and unobtrusively administered in laboratory settings (Blascovich et al. 2011).

#### Arousal and Pleasantness

An adapted version of the affect grid was used to measure the two dimensions of core affect, *arousal* and *pleasantness*, which have been linked to performance in motor and cognitive tasks (Russell et al. 1989). Participants were asked to report their perceived arousal levels on a Likert scale ranging from 0 (*sleepiness*) to 10 (*highly aroused*), and to report on “How pleasant you believe the task is?” on a Likert scale ranging from 0 (*not pleasant*) to 10 (*very pleasant*).

#### Attentional States

Attention influences the execution of motor skills, including video game playing (Gray 2017). Participants were asked to report their *attentional states* on a Likert scale ranging from 0 (*distracted/unable to focus*) to 10 (*complete focus on task*), congruent with previous research in applied psychology (Basevitch et al. 2011).

#### Self-efficacy and Others’ Efficacy

Efficacy beliefs are strong predictors of performance in individual and team tasks (Bandura 1997). As such, participants were asked to rate “The belief you have in your own skills/abilities to win the match” and to state their *others’ efficacy* by responding to the statement “The belief you have in your teammates abilities/skills to help you win the match” on a Likert scale ranging from 0 (*no belief*) to 10 (*complete belief*). Both questions were designed in line with Bandura’s (2006) recommendation for the development of efficacy measures.

### Communication Data

Throughout the experimental task, participants were asked to wear a sociometric badge (Sociometric Solutions 2013, USA), which has been shown to reliably record communication metrics over time (Kim et al. 2012). Specifically, the badges recorded, in an arbitrary unit, the amount of *Total Silence* (i.e., no spoken communication), *Total Speaking* (i.e., spoken communication), *Listening* (i.e., only one participant speaking) and *Overlap* (i.e., one participant talking over the other).

### Cardiovascular Data

A Polar H10 chest strap (Polar Electro QY 2017) was used to collect the participants’ cardiovascular responses, namely *heart rate* (HR) and the Root Mean Squared of Successive Differences (RMSSD), which is a *heart rate variability* (HRV) index. RMSSD is the main time-domain HRV index because it reflects beat-to-beat acute stress changes in HR (Laborde et al. 2017).

### Experimental Task and Procedures

Participants were briefed on the study and written consent was obtained. Each participant was paired with another participant, whom they had not met before (i.e., zero-acquaintance tenure), to form a dyadic team. As recommended in the literature (Blascovich et al. 2011), a baseline assessment during which the participants sat in silence for two minutes was recorded to ensure all equipment were working properly and that the participants’ physiological data were within normal ranges. The participants then played the video game FIFA 17 using the XBOX ONE console system. The video game was played on a 44.17 × 23.77-inch screen, which was distanced two meters from the participants. Each dyad played three 10 min games (i.e., 5 min per half) against the computer. All games were played with a pre-determined “professional difficulty level” with the computer playing as Barcelona and the participants as Real Madrid. During each game, both participants had their communication patterns (i.e., *Total Speaking, Total Silence, Listening, Overlap*) and physiological responses (i.e., *HR* and *HRV*) monitored. Furthermore, each participant was asked to report on their perceived psychological states (i.e., *Arousal*, *Pleasantness, Attentional States, Self*-*Efficacy* and *Others’ Efficacy*) at baseline, before, at half-time and after each game. The communication data was time-stamped to allow for posterior analysis. The data collection procedure lasted approximately 2 h.

### Data Analysis

The unit of analysis consisted of one entire game. As such, mean scores for all dyads in each game (i.e., Game 1, Game 2, and Game 3) and across all variables were computed. As detailed elsewhere, means scores allow for a more reliable “whole team estimate” of the variables of interest (Thorson et al. 2018). The communication metrics were exported using the Sociometric Lab software (Version 1.41, USA), and *HR* and *HRV* were both filtered and exported from Kubios (Version 3.1). All data were inputted into IBM Statistics SPSS 24.

## Results

Single effects repeated measures ANOVA with a Greenhouse–Geisser correction were computed for all variables and, where applicable, Bonferroni corrections were used for all post-hoc comparisons. Noteworthy, repeated measures ANOVA is a robust and recommended approach for the analysis of team data when an equal time interval and equal number of data points are taken into consideration (Raudenbush 2004; Shin 2009). Cohen’s guidelines (2012) were used to classify effect sizes as small (*d* = .20), medium (*d* = .50) and large (*d* ≥ .80). Congruent with current standards of reporting (see Appelbaum et al. 2018), in addition to *p*-values, mean, standard deviation and effect size metrics for all variables are reported in Table 1. All moderate-to-large statistical effects (*d* > .50) observed across studies and variables are visually depicted in figures and graphs throughout the manuscript.

**Table 1:**
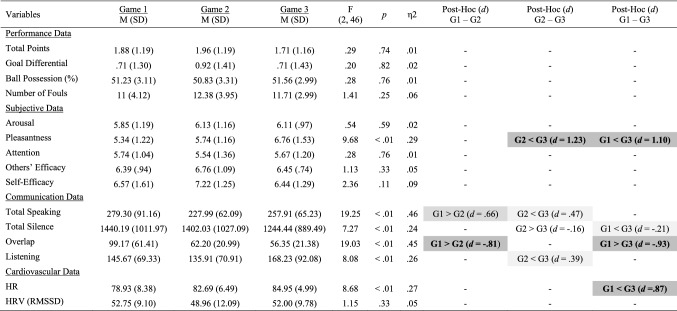
Performance, subjective, communication and cardiovascular data across games

Significant small effects (.20 ≤ *d* > .50) are highlighted in light grey, medium effects (.50 ≤ *d* < .80) are in darker grey, and large effects (*d* ≥ .80) are bolded and highlighted in the darkest grey colour

### Performance and Subjective Data

**Fig. 1:**
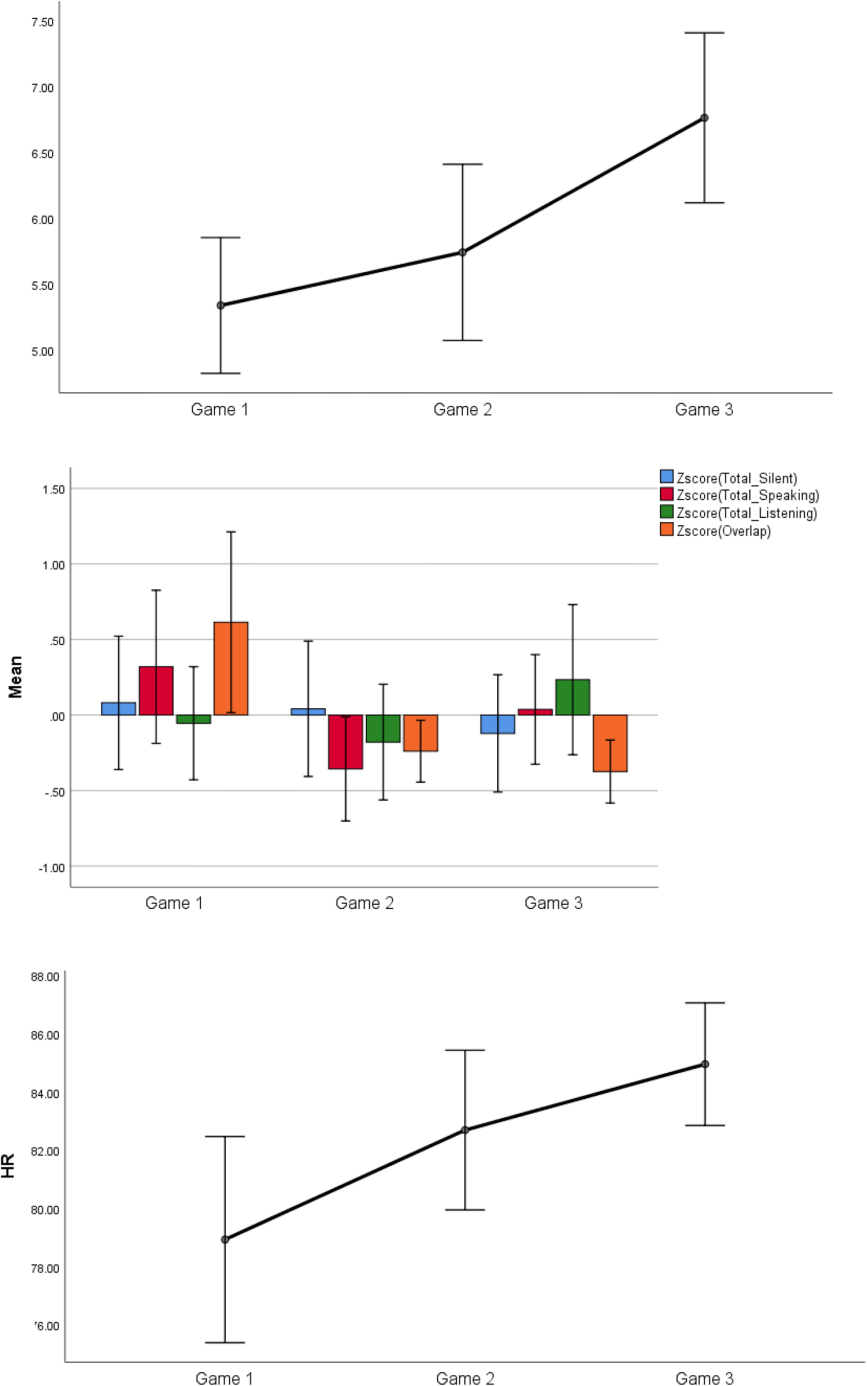
Changes in pleasantness (upper panel), communication patterns (middle panel) and HR (lower panel) across games

No statistical effects were observed for all performance and subjective variables, except *Pleasantness*, which increased to a large extent from Game 1 to Game 3 (*p* < .01; *d* = 1.10), and from Game 2 to Game 3 (*p* < .01; *d* = 1.23), but did not statistically differ from Game 1 to Game 2 (Fig. 1, upper panel).

### Communication Data

Standardized changes in all communication variables are illustrated in Fig. 1 (middle panel). *Total Silence* decreased slightly from Game 1 to Game 3 (*p* < .01; *d* = − .21), and from Game 2 to Game 3 (*p* < .01; *d* = − .16). *Total Speaking* decreased to a large extent from Game 1 to Game 2 (*p* < .01; *d* = .66) and increased to a moderate extent from Game 2 to Game 3 (*p* < .01; *d* = .47). *Listening* increased to a moderate extent from Game 2 to Game 3 (*p* < .01; *d* = .39). *Overlap* decreased to a large extent from Game 1 to Game 2 (*p* < .01; *d* = − .81) and from Game 1 to Game 3 (*p* < .01; *d* = -.93).

### Cardiovascular Data

*HR* increased greatly from Game 1 to Game 3 (*p* < .01; *d* = .87; Fig. 1, lower panel). No statistical changes in *HRV* were observed.

## Discussion

For brevity, in this section we highlight the significant effects observed in this study for the communication variables, pleasantness and HR. We elaborate upon the non-significant effects observed for all other variables in the General Discussion at the end.

### Changes in Communication Patterns, Pleasantness and HR Over Time

We observed changes in the communication patterns among teammates over time. By the third game, teammates were listening more and talking less, and consequently turn-taking was more efficient (i.e., less overlap). These findings coincide with research suggesting that as teammates practice together turn-taking improves and communication shifts from overt to covert means, which signals overall coordination efficiency gains (Boulton and Cole 2016; LeCouteur and Feo 2011; Westli et al. 2010). Moreover, the observed increase in pleasantness is in line with previous research showing that core pleasantness levels fluctuate greatly over time in both individual and group tasks (di Fronso et al. 2020). More specifically, the observed increase in pleasantness over time reinforces research suggesting that as teams develop, teammates move out of the so-called “storm stage”, with the result being more positive affect for the individuals and the emergence of a sense of “we/us” at the group-level of analysis (Bonebright 2010). Positive core affect has also been shown to increase over time. Finally, we suggest that the increase in pleasantness over time might be linked to the increase in HR from Game 1 to Game 3, as positive affective valences (e.g., happiness, excitement) elicit parasympathetic withdraw and increase adrenaline levels (Laborde et al. 2017). Alternatively, this increase in HR might be due to fatigue, even though participants were given breaks between games.

## Study 2

We expanded Study 1 by incorporating brain imaging methods to explore neural marks of coordination cost. Rather than merely replicating Study 1, we reasoned that it was important to first examine immediate (acute) performance and psycho-bio-social responses to team settings. The specific aim was to explore differences in performance and individuals’ perceived psychological states, cardiovascular responses and absolute brain power (i.e., alpha, beta and theta) in a single video game match across two conditions, namely individual playing and team dyadic playing. We expected that the dyadic condition would lead to lower performance and elicit more negative affective states and efficacy beliefs akin to the idea of coordination cost (Becker and Murphy 1992; Eccles 2010) and research on the initial stages of team development (for a review see Bonebright 2010). Further, we anticipated that the dyadic condition would require higher attention, cardiovascular stress, and brain power across frequency bands. In the early stages of team development, several team processes that antecede and reinforce team coordination (e.g., cohesion, collective efficacy; see Filho et al. 2015b; Filho 2019) are not developed yet, and thus individuals might exert more psycho-bio-social resources (the so-called coordination cost) to complete teamwork.

## Methods

### Participants

New participants were recruited for this study. An a priori power analysis (*d* = .50; 1 − β = .95; α = .05) based on previous research in performance psychology (Bertollo et al. 2015) indicated that 12 participants were needed to detect a moderate-to-large effect on the variables of interest. We chose a moderate-to-large effect size because we were interested in non-trivial effects. Furthermore, our target sample involved skilled gamers, and the recruitment of skilled individuals across domains of human performance is a challenging task (Ericsson 1998). Accordingly, 12 individuals and one confederate participated in Study 2. Participants were assembled into 12 dyads, with the confederate being kept as a constant and thus playing in all dyads. All participants were in their twenties (M = 21.69, SD = 2.46), male, and had at least 30 h of experience playing FIFA 17. The confederate was 20 years old, had two years of experience playing FIFA 17, and reported practicing for approximately 2 h a week. He was briefed on the methodology but was not aware of the overarching purpose of the study.

### Measures

#### Performance Data

The same performance measures were used as in Study 1, namely, *Total Points*, *Ball Possession*, *Goal Differential*, and *Number of Fouls*.

#### Subjective Data

The same subjective reports were collected as in Study 1 (i.e., *Arousal*, *Pleasantness*, *Attentional States* and *Self-Efficacy*), except for *Others’ Efficacy* as data from the confederate was not considered in the analysis.

#### Cardiovascular Data

Each active player (AP) had his *HR* and *HRV* data collected in the same manner as in Study 1.

#### EEG Data

EEG data was continuously recorded throughout the experimental task using the Nexus-32 biofeedback system (Mind Media B.V., Netherlands). *Alpha, Beta* and *Theta Absolute Power* were measured in microvolts squared (μV^2^) across 21 different channels at a sampling frequency of 256 Hz. The 21 Ag/AgCl electrodes were positioned over the scalp according to the 10/20 system (Oostenveld and Praamstra 2001). EEG signals were recorded with the ground electrode in AFz positioned between Fpz and Fz. Low independence values were kept during the data collection (Z < 5 kO).

### Experimental Task and Procedures

Before data collection, the goals and methods of the study were explained to the participants, and written consent was obtained. Participants were then placed into a dyad with the confederate. The experimental task consisted of two conditions (i.e., individual and dyad) in which the participants played FIFA 17 using the XBOX ONE console system. Each experimental condition was preceded by a baseline assessment, during which the AP sat in silence for two minutes with his eyes open and then for an additional two minutes with his eyes closed, to ensure the equipment was working properly.

The AP played with the confederate (dyad condition) and without the confederate (individual condition). Each game lasted 10 min (i.e., 5 min per half) and was played using the same settings described in Study 1. Importantly, to minimize movement artifacts with the EEG data, no communication was allowed before, during, or after either condition. Also, akin to similar research (see Yuvaraj et al. 2014), the participants were given a five-minute break between games to minimize potential feelings of fatigue.

For the individual condition, the AP played a game of FIFA 17 against the computer by themselves. For the dyad condition, the AP played together with the confederate against the computer using the same pre-determined teams and pre-established difficulty settings as explained in Study 1. The two experimental conditions were counterbalanced. During both games, the AP had his cardiovascular and EEG activity recorded. Furthermore, the AP was asked to report on his perceived psychological states before, at half-time and after each game. The confederate was also asked to report on his psychological states during the dyad condition at the same intervals, but his data was not integrated in the data analysis. The entire data collection procedure lasted about 2 h.

### Data Analysis

As with Study 1, the unit of analysis was one entire game. The subjective and cardiovascular data was treated in the same way as in Study 1. All EEG data was visually inspected to remove artefacts, band-pass filtered and exported using the BioTrace+software built-in function. Event markers were used to segment the data into 6 s epochs akin to previous research suggesting that 6–15 s time windows should be used in the processing of bio-signal data (Kim et al. 2004; Yuvaraj et al. 2014). These segments were exported to IBM Statistics SPSS 24, averaged across each game, and then descriptively and inferentially analyzed.

## Results

Mean and standard deviations values, Cohen’s *d* effect size differences, power, and *p*-values for the performance, subjective, cardiovascular and EEG measures are reported in Tables 2, 3, 4 and 5.

**Table 2:**
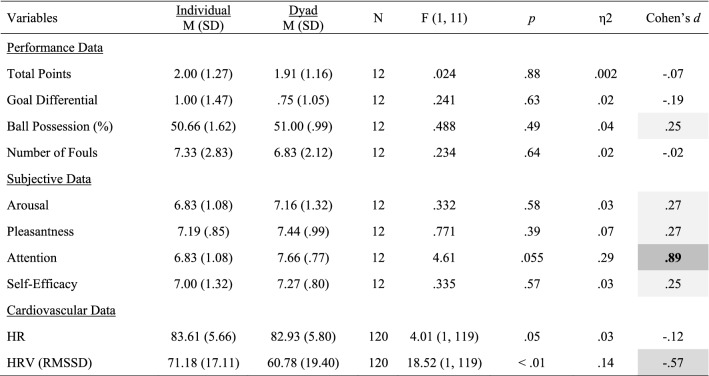
Performance, subjective and cardiovascular data for the individual and dyad conditions

Significant small effects (.20 ≤ *d* > .50) are highlighted in light grey, medium effects (.50 ≤ *d* < .80) are in darker grey, and large effects (*d* ≥ .80) are bolded and highlighted in the darkest grey colour

**Table 3:**
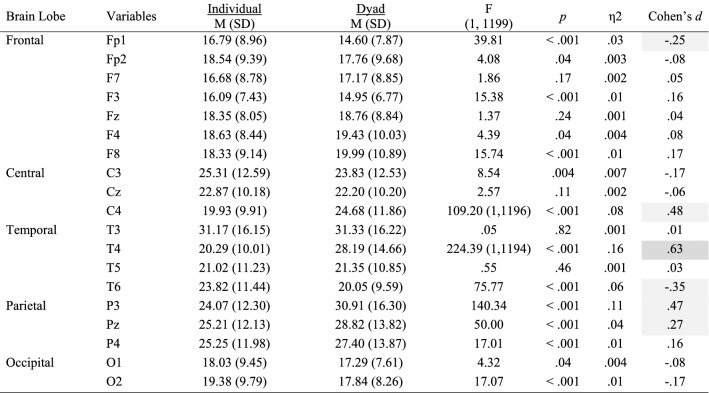
Alpha power for the individual and dyad conditions

Significant small effects (.20 ≤ *d* > .50) are highlighted in light grey, medium effects (.50 ≤ *d* < .80) are in darker grey, and large effects (*d* ≥ .80) are bolded and highlighted in the darkest grey colour

**Table 4:**
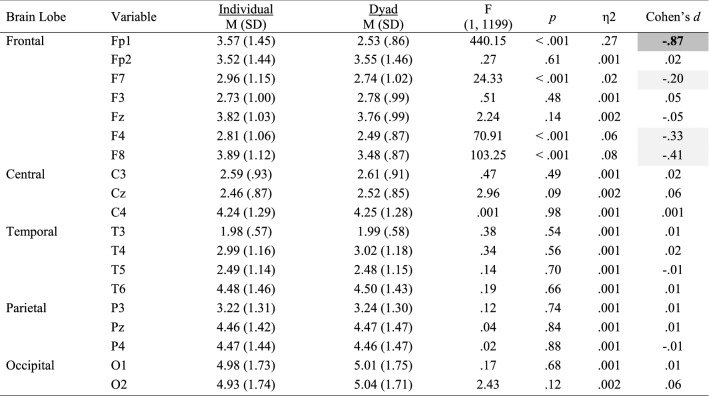
Beta power (μV^2^) for the individual and dyad conditions

Significant small effects (.20 ≤ *d* > .50) are highlighted in light grey, medium effects (.50 ≤ *d* < .80) are in darker grey, and large effects (*d* ≥ .80) are bolded and highlighted in the darkest grey colour

**Table 5:**
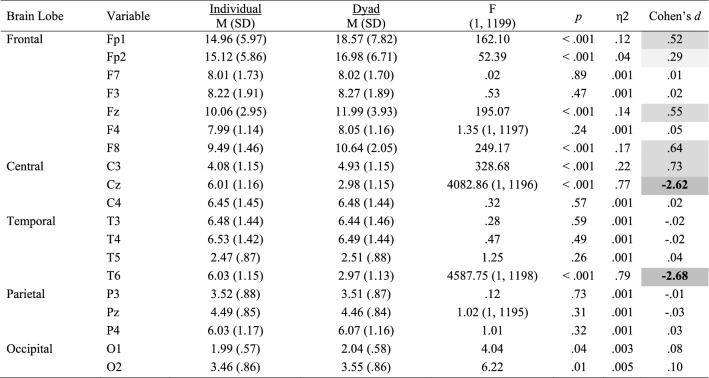
Theta power (μV^2^) for the individual and dyad conditions

Significant small effects (.20 ≤ *d* > .50) are highlighted in light grey, medium effects (.50 ≤ *d* < .80) are in darker grey, and large effects (*d* ≥ .80) are bolded and highlighted in the darkest grey colour

### Performance and Subjective Data

**Fig. 2:**
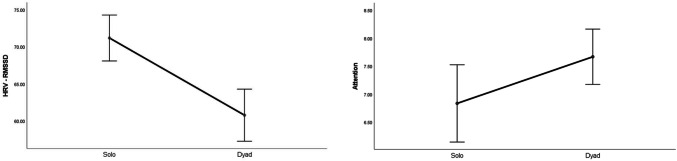
Significant changes of moderate-to-large magnitude (d ≥ .50) in attentional levels (left panel) and HRV-RMSSD (right panel) between the individual and the dyadic conditions

*Attention* increased to a large extent in the dyadic condition (*p* = .055; *d* = .89; see Fig. 2, left panel). No other statistical differences were observed for all performance and subjective variables.

### Cardiovascular Data

Statistical differences were observed for *HR* and *HRV*, with magnitude effect size analyses suggesting that *HR* decreased slightly (*d* = − .12), whereas *HRV* decreased moderately (*d* = − .57; Fig. 2, right panel) in the dyad condition.

### EEG Data

**Fig. 3:**
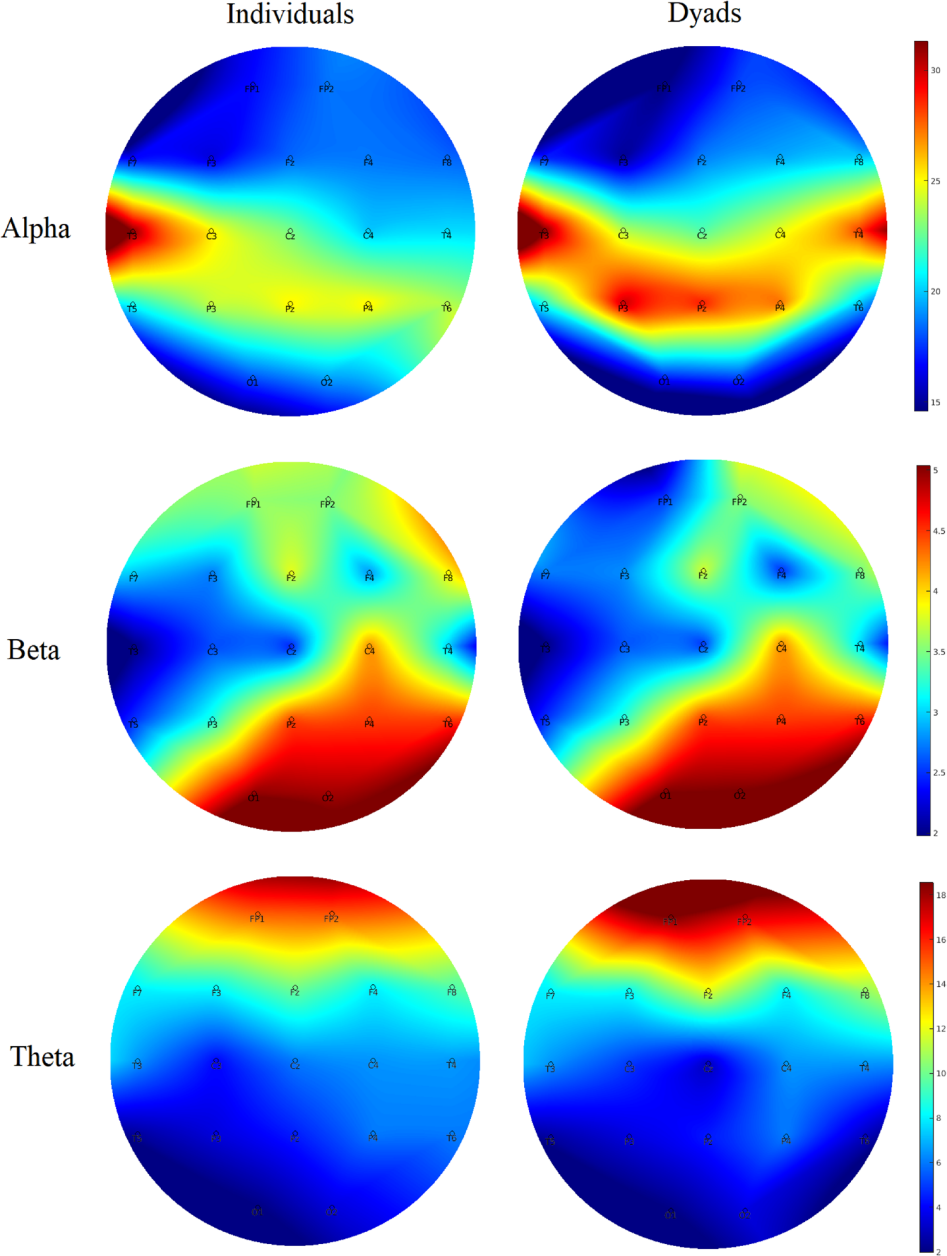
Heat map for absolute power (μV^2^) of alpha (upper panel), beta (middle panel) and theta (lower panel) for the individual and dyad condition showing an overall higher pattern of activation in the dyadic condition across frequency bands. The range set for alpha (14.60–31.33), beta (1.98–5.04) and theta power (1.99–18.57) were established based on the observed values in the data set

**Fig. 4:**
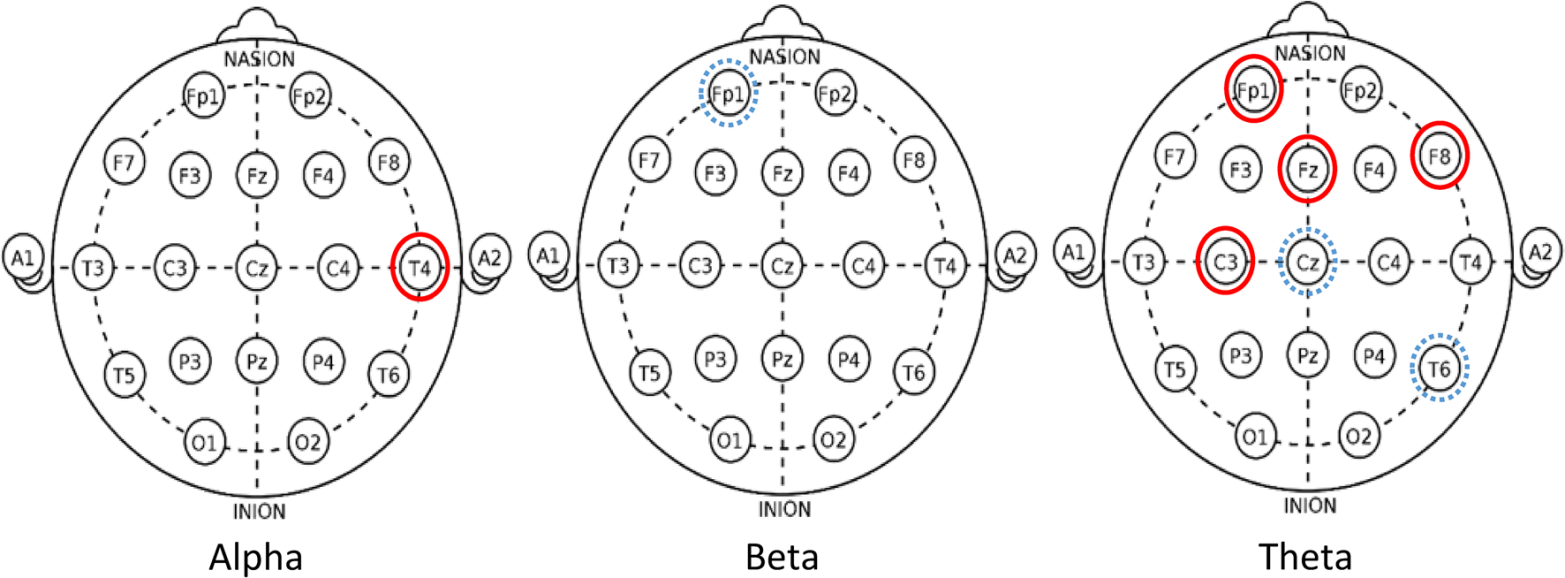
Significant changes of moderate-to-large magnitude (d ≥ .50) in alpha (left panel), beta (middle panel) and theta (right panel). Solid (red) lines indicate an increase in the dyad condition. Dashed (blue) lines indicate a decrease in the dyad condition

Inferential and descriptive statistics for alpha, beta and theta absolute power are reported in Tables 3, 4 and 5, respectively. Topographic head models were generated based on the raw absolute power for each frequency band (see Fig. 3), revealing that, for the most part, similar brain areas where activated in both conditions; however, the intensity of this activation differed. Indeed, changes of small-to-medium magnitude (.20 < *d* > .50) were observed in all frequency bands, and changes of moderate-to-large magnitude (*d* ≥ .50) are illustrated in Fig. 4. Together, these findings suggest that individual work and teamwork hinge on different neural activation patterns as elaborated upon in Discussion.

## Discussion

In this section, we highlight the observed significant changes in attention, HRV and brain rhythms. We elaborate upon the non-significant findings observed for all other variables in the General Discussion.

### Changes in Attention, HRV, and Brain Rhythms in Teamwork

Congruent with our expectations grounded on the aforementioned notion of “coordination cost”, we observed an increase of large magnitude in perceived attention for the dyadic condition. We suggest that greater focus, rather than diffused attention, is needed for teamwork because teamwork relies on division of labour (Eccles 2010; Gable et al. 2018; Stander 1992), and thus individual team members must pay greater attention to fewer things. In the dyadic condition, individuals are responsible for specific sub-tasks (i.e., adaptive specialization), which in turn allows for a greater focus as it decreases the cost of switching between tasks, and ultimately increases individual efficiency. This explanation is congruent with the large decrease in HRV and the large increase of frontal theta brain activity (Fp1, Fz, F8) observed in the dyadic condition, as such general cardiovascular and neural patterns signal deep (“flow-like”) concentration in the execution of both motor and cognitive tasks (Katahira et al. 2018; Laborde et al. 2017). Participants’ higher perceived levels of attention also coincides with the large increase of alpha activity and the large decrease of theta activity observed in the dyadic condition for T4 and T6, respectively. Specifically, increased alpha activity in T4 and decreased theta activity in T6 have been associated with internally-focused attention during task execution (Benedek et al. 2014), and the mnemonic encoding of new information (Fellner et al. 2016), respectively. Furthermore, the large increase of theta activity in C3, an area related to sensory-motor specialization (see Strack et al. 2011), suggests that the AP was making a conscious effort to assimilate the new sensory-motor demands imposed by the dyadic condition.

Whereas the dyadic task required greater focused attention as indicated by the increased theta activity in C3 and frontal areas, it required less motor activity and decision-making processing, as indicated by the large decreases observed in theta power for the CZ and beta power for the Fp1 sites, respectively. Thus, in teams, more focused attention for the learning and execution of a specialized task is needed but, in turn, less decision-making processing and motor effort is required. Therefore, if for a given task teamwork is advantageous by nature, such advantage might not be clear at the initial stages of team development, as this study shows and previous research has documented (Bonebright 2010; Filho et al. 2015a, b; Filho 2019; Gabelica et al. 2016) because other team properties that precede coordination are not well-developed yet.

## Study 3

To expand upon Study 1 and Study 2, we compared team performance and individuals’ perceived psychological states, cardiovascular responses and alpha, beta and theta power over three games. As teammates develop shared and complementary knowledge over time (see Filho and Rettig 2018; Filho and Tenenbaum 2020; Mohammed et al. 2010; 2017), we expected positive increases in performance, core affect and efficacy beliefs from Game 1 to Game 3. Furthermore, due to adaptive task specialization (i.e., teamwork saves individuals’ energy through division of labour) that comes with team development over time (Duarte et al. 2012; Eccles 2010), we expected a decrease in attention, cardiovascular responses, and absolute brain power from Game 1 to Game 3.

## Methods

### Participants

New participants were recruited for this study. An a priori power analysis (*d* = .50; 1 − β = .95; α = .05) was used to establish the minimum sample size (N = 12) needed to detect a moderate to strong effect size on the variables of interest. All participants (N = 24) were in their twenties (M = 21.79, SD = 1.74), had at least 30 h of experience playing FIFA 17, and were assembled into 12 dyads.

### Measures

The same performance, subjective, cardiovascular and EEG data collected in Study 2 were gathered, namely: *Total Points, Ball Possession, Goal Differential, Number of Fouls, Arousal, Pleasantness, Attentional States, Self-Efficacy, Others’ Efficacy, HR, HRV, and Alpha, Beta* and *Theta Absolute Power.*

### Experimental Task, Procedures, and Data Analysis

In Study 3 participants played three consecutive games against the computer, and there was no confederate. One participant from each dyad was randomly chosen to be the AP, from who physiological and EEG recordings were taken during the experiment, while the other participant (“Participant B”) only responded to the subjective reports. The data was analyzed following the same step-by-step procedure used for Study 2.

## Results

### Performance and Subjective Data

**Fig. 5:**
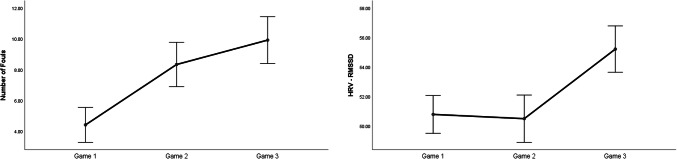
Significant changes of moderate-to-large magnitude (d ≥ .50) in the number of fouls and HRV (RMSSD) from game 1 to game 3

*Number of Fouls* increased greatly from Game 1 to Game 2 (*p* =  < .01; *d* = .92) and from Game 1 to Game 3 (*p* ≤ .01; *d* = 2.61; see Fig. 5, right panel). No other statistical differences were observed (see Table 6).

**Table 6:**
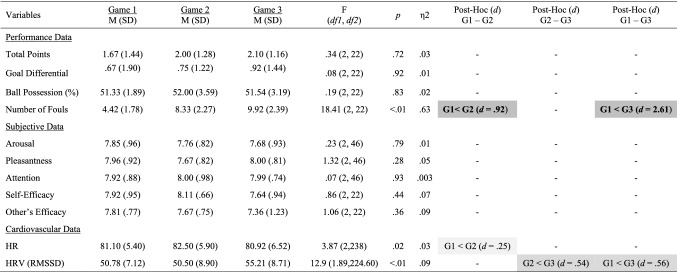
Performance, subjective and cardiovascular data for game 1, game 2, and game 3

Significant small effects (.20 ≤ *d* > .50) are highlighted in light grey, medium effects (.50 ≤ *d* < .80) are in darker grey, and large effects (*d* ≥ .80) are bolded and highlighted in the darkest grey colour

### Cardiovascular Data

*HR* increased from Game 1 to Game 2 (*p* = .02; *d* = .25). HRV increased to a moderate extent from Game 1 to Game 3 (*p* ≤ .01; *d* = .56), and from Game 2 to Game 3 (*p* ≤ .01; *d* = .54; see Fig. 5, right panel).

### EEG Data

**Fig. 6:**
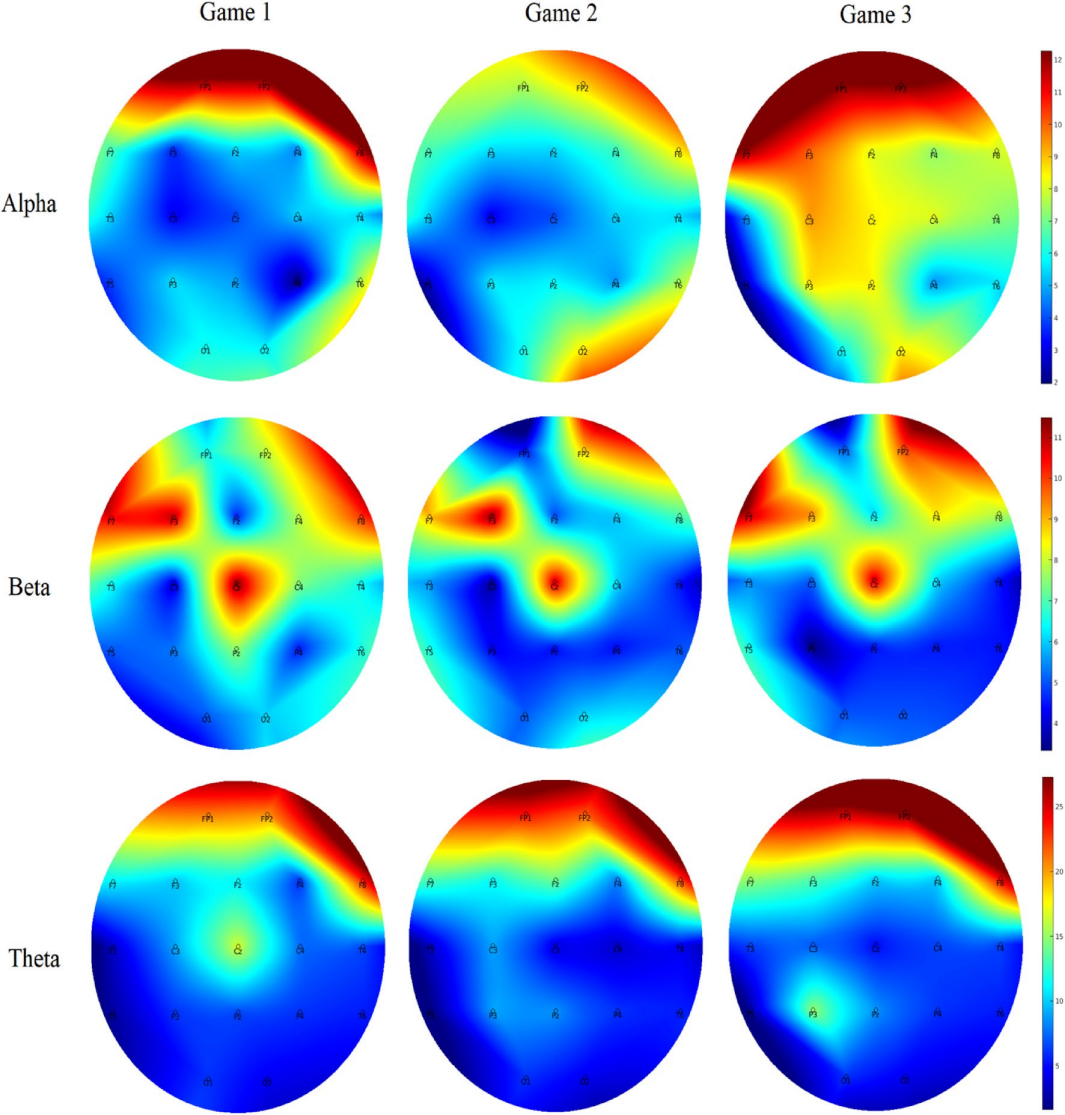
Heat map for absolute power of alpha (upper panel), beta (middle panel) and theta (lower panel) for games 1–3 showing changes in the activation pattern over time across frequency bands. The range set for alpha (1.97–12.28), beta (3.34–11.49) and theta power (1.64–27.31) were established based on the observed values in the data set

**Fig. 7:**
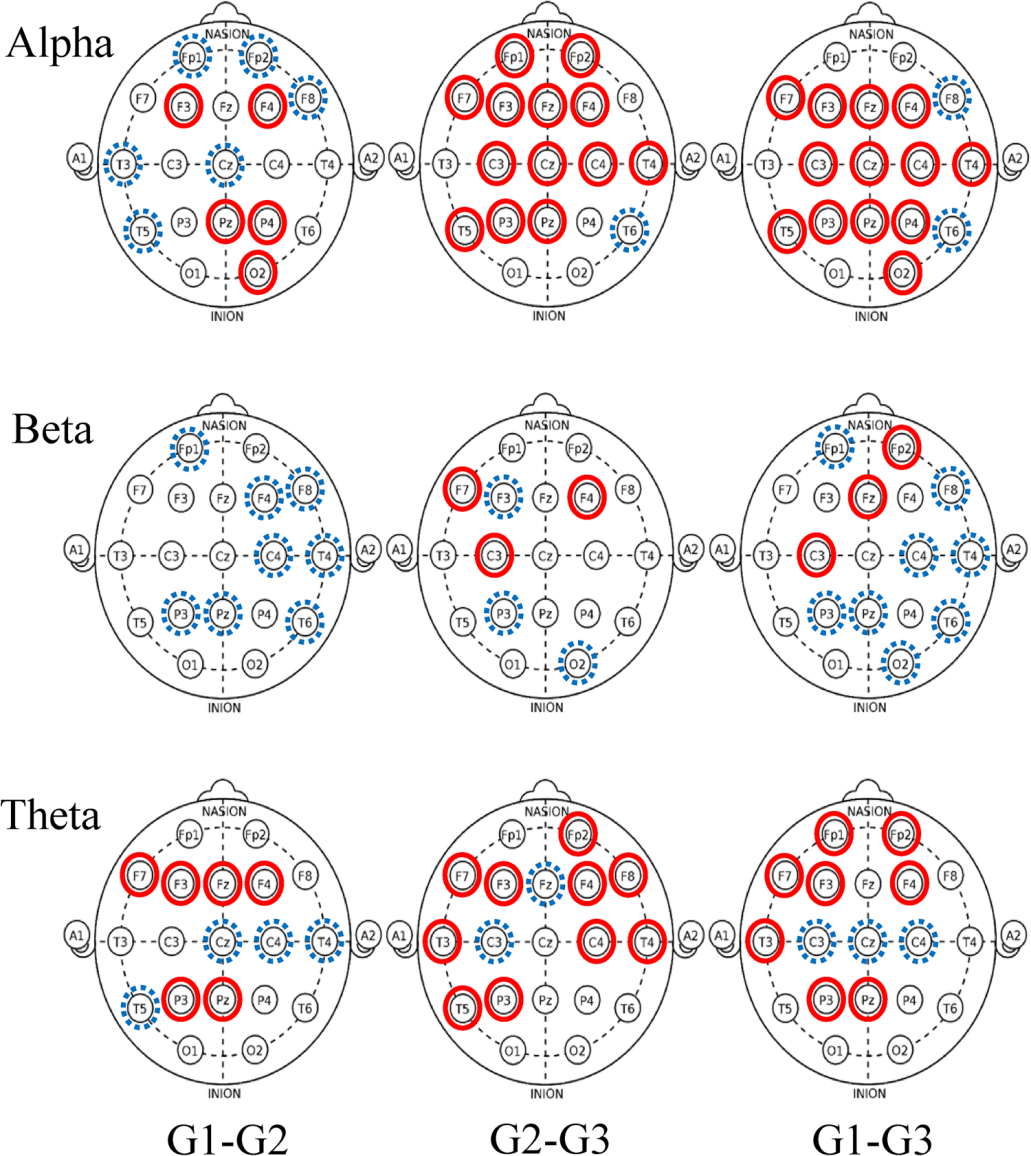
Significant changes of moderate-to-large magnitude (d ≥ .50) in alpha (upper panel), beta (middle panel) and theta (lower panel) brain power from game 1 to game 3. Solid (red) lines indicate an increase in absolute power and dashed (blue) lines indicate a decrease in absolute power

Inferential and descriptive statistics for alpha, beta, and theta absolute power are reported in Tables 7, 8 and 9, respectively. Topographic head models (see Fig. 6) revealed changes in absolute brain power from Game 1 to Game 3 across all frequency bands. Similar to Study 2, changes of small-to-medium magnitude (.20 < *d* > .50) were observed in all frequency ranges across all games. Changes of moderate-to-large magnitude (*d* ≥ .50) are illustrated in Fig. 7. Collectively, these findings suggest that over time, as team members learn to work in teams, individuals’ brain states move towards a neural efficiency state, as elaborated upon next.

**Table 7:**
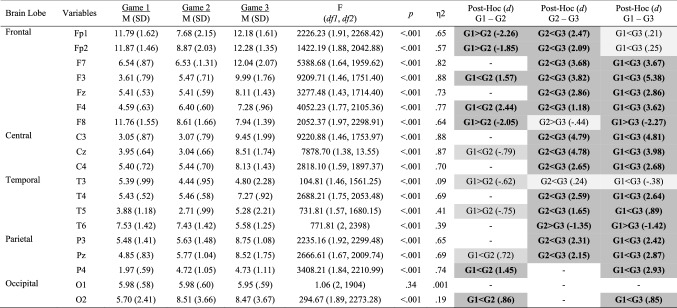
Alpha power (μV^2^) across game 1, game 2 and game 3

Significant small effects (.20 ≤ *d* > .50) are highlighted in light grey, medium effects (.50 ≤ *d* < .80) are in darker grey, and large effects (*d* ≥ .80) are bolded and highlighted in the darkest grey colour

**Table 8:**
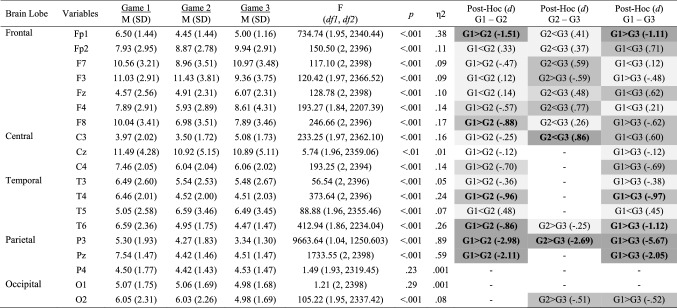
Beta power (μV^2^) across game 1, game 2 and game 3

Significant small effects (.20 ≤ *d* > .50) are highlighted in light grey, medium effects (.50 ≤ *d* < .80) are in darker grey, and large effects (*d* ≥ .80) are bolded and highlighted in the darkest grey colour

**Table 9:**
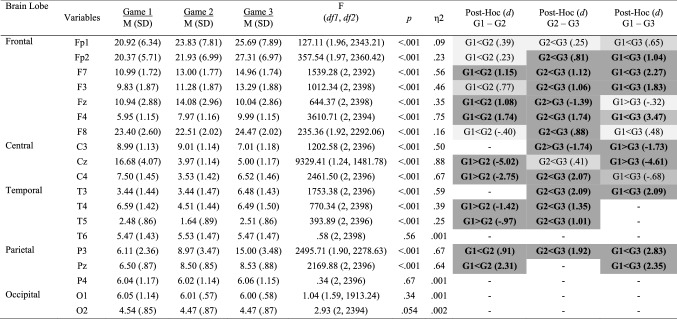
Theta power (μV^2^) across game 1, game 2 and game 3

Significant small effects (.20 ≤ *d* > .50) are highlighted in light grey, medium effects (.50 ≤ *d* < .80) are in darker grey, and large effects (*d* ≥ .80) are bolded and highlighted in the darkest grey colour

## Discussion

In this section, we highlight the significant effects observed in this study for performance data (number of fouls), cardiovascular responses and brain rhythms. We comment on the non-significant findings observed for all other variables in the General Discussion.

### Changes in Performance (Number of Fouls), Cardiovascular Responses, and Brain Rhythms Over Time

We observed a large increase in the number of fouls over time. We suggest that this increase is because teammates were not allowed to communicate, which likely decreased coordination and increased frustration, ultimately leading to instrumental aggression in the video game play (see the *frustration-aggression* hypothesis in Berkowitz 1989). Although the amount of spoken communication tends to decrease over time, language evolved in the natural world to allow for improved team coordination and super-efficiency, as research across domains has consistently shown (Anderson and Franks 2001; Boulton and Cole 2016; Duarte et al. 2012; LeCouteur and Feo 2011; Westli et al. 2010).

Moreover, we observed a decrease in HRV in Game 3, compared with Game 1 and Game 2, suggesting that less psycho-bio-social stress and mental workload was required in Game 3, likely because teammates developed shared and complementary knowledge and engaged in division of labour. Importantly, we observed an increase in HR from Game 1 to Game 2. Similar to our interpretation for the findings for Study 1, we argue that this increase might reflect fatigue or parasympathetic withdraw. Notably, this increase cannot be attributed to the fact that individual work is more demanding than teamwork, given that in Study 2 we observed a decreased in HR in the dyadic condition.

In Game 3, we also observed a global increase in alpha activity and decrease in beta cortical activity, which are indicative of less brain “busy-ness” and skilled motor performance, akin to the *neural efficiency hypothesis* (see Bertollo et al. 2016; Grabner et al. 2006; Pacheco 2016). In this regard, previous research suggests that peaks of alpha activity (more relaxation) and less beta power (increased automaticity) are observed across the whole brain as individuals become more proficient in a given task and/or are subjected to less work overload (Bertollo et al. 2016; Pacheco 2016). We also observed large increases of theta power activity across the whole brain from Game 1 to Game 2 to Game 3, further suggesting that more focused attention is needed over time likely because teammates develop task and team-related knowledge (Cooke et al. 2000; Filho and Rettig 2018; Filho and Tenenbaum 2020; Mohammed et al. 2010, 2017), which form the basis for team coordination.

## General Discussion

We expected to observe positive changes in communication patterns (i.e., speaking, silence, listening, and overlap), core affect (i.e., arousal and pleasantness), efficacy beliefs (i.e., self and other’s), attentional levels, cardiovascular responses (i.e., HR and HRV), and brain rhythms (i.e., alpha, beta and theta absolute power) over time (Study 1 and Study 3), and when comparing individual to team work (Study 2), akin to an evolutionary perspective on teamwork.

Contrary to our expectations, we did not observe a positive change in individuals’ arousal levels, efficacy beliefs, and performance variables across studies. All studies were conducted in 1 day and over a maximum of three 10-min video game matches, and thus we might not have been able to capture changes in efficacy beliefs and performance, as these take time to develop in both individuals and team settings (see Bandura 1997). Arousal levels, on the other hand, have been shown to be highly idiosyncratic as discussed in the Individual Zones of Optimal Functioning framework (Hanin 2000).

For the most part, however, the generally expected pattern of results was observed. The pattern is complex, as discussed throughout, and akin to the notion that team processes and individuals’ that psycho-bio-social states share a many-to-many basis relationship and thus should be analysed by the whole rather than by the parts (Cacioppo et al. 2007). More specifically, congruent with the aforementioned research on team dynamics and coordination, our findings suggest that over time teammates develop more efficient communication patterns, characterized by less talking, more listening and less overlap (Study 1). As such, precluding teammates from communicating freely has potential implications for aggressive behaviour and performance (i.e., increased number of fouls) in team settings, as observed in Study 3. Moreover, as observed in Study 2, an increased focused attention, likely reflecting adaptive task specialization, is the cost of teamwork in the early stages of team development. Finally, over time individuals experience whole brain functional changes across frequency bands and an increase in HRV, highlighting that less mental overload (neural efficiency) and less cardiovascular stress (psychomotor efficiency) are the benefits of teamwork (Study 3).

## Limitations and Future Research

Across studies our focus on skilled individuals precluded us from gathering a larger sample. Novice video game players would likely confound the findings as individuals’ skill-level is implicated in individual and group psychology (Ericsson 1998; Filho et al. 2015a, b). Larger sample sizes are warranted in future research if we are to model the relationship among individuals’ psycho-bio-social states and team processes and outcomes using multi-level statistical methods. Moreover, our study was descriptive in nature. Whereas descriptive experimental research is needed to reach what Chomsky (1965) has called “descriptive adequacy” in theoretical reasoning, future research is needed to test clear means-ends relations among (i.e., explanatory adequacy) variables of interest. To this point, in Study 3, we have speculated that lack of communication lead to poor coordination which then lead to aggressive behaviour; this input-throughout-output relation can be tested in future research.

In Study 1, we used sociometric badges to capture the meta-features of the participants’ communication exchanges. We choose not to video-record the participants’ communication exchanges partially to prevent EEG artifacts, because this has been done before (e.g., LeCouteur and Feo 2011), and to avoid them to become self-conscious and change their most natural behaviour. Future research recording participants verbal and non-verbal communication, while monitoring their psycho-bio-social states and potentially brain waves (if movement artifacts can be accounted for with portable EEG systems) can add complementary and potentially alternative responses to our questions on coordination cost and super-efficiency in teamwork.

Finally, in Study 2 and Study 3, we only looked at data from one randomly chosen participant due to material constraints. Multi-person peripheral physiological monitoring in general, and hyper-brain studies in particular, are warranted to advance understanding of the neural markers of team coordination and other team processes (see Filho et al. 2015a, 2016, 2017; Sänger et al. 2012, 2013; Stone et al. 2019). To this extent, alpha, beta and theta activity is related to several cognitive processes (e.g., selective attention; sensorimotor integration; drowsiness; see Başar and Güntekin 2012; Bazanova and Vernon 2014; Cheron et al. 2016) and hyper-brain studies are needed to further clarify the neural markers of team coordination.

## Conclusions

We advanced previous research by exploring team coordination dynamics through a multimodal methodological approach, and particularly by incorporating brain imaging methods to shed light on the notion of coordination cost and super-efficiency in teamwork. Overall, our findings suggest that there is a trade-off between coordination cost and super-efficiency. First, teamwork might increase individuals’ attentional focus and global neural efficiency by decreasing the costs of switching between tasks. More generally, for some tasks individual work might be better because one must pay less focused attention to specific roles, whereas for other tasks teamwork might be better because there is less motor effort and decision fatigue involved. At the earliest stages of team development, the trade-off between coordination cost and super-efficiency is less clear. It is only over time (perhaps long periods of evolution) that the so-called “balance of nature”, at the core of Darwinism, becomes clear. In light of these findings, scholars and practitioners should address team dynamics over time through different means, including interventions targeting communication dynamics, attentional focus and core affect, cardiovascular responses, and neurofeedback training aimed at different brain rhythms.
